# Genome-wide epigenetic variation among ash trees differing in susceptibility to a fungal disease

**DOI:** 10.1186/s12864-018-4874-8

**Published:** 2018-06-28

**Authors:** Elizabeth S. A. Sollars, Richard J. A. Buggs

**Affiliations:** 1 School of Biological and Chemical Sciences, Queen Mary University of London, Mile End Road, London, E1 4NS UK; 2 Jodrell Laboratory, Royal Botanic Gardens Kew, Richmond, Surrey TW9 3AB UK

**Keywords:** *Fraxinus excelsior*, Ash dieback, Epigenetics, DNA methylation, Whole genome duplication, Disease susceptibility

## Abstract

**Background:**

European ash trees (*Fraxinus excelsior*) are currently threatened by ash dieback (ADB) caused by the fungus *Hymenoscyphus fraxineus* but a small percentage of the population possesses natural low susceptibility. The genome of a European ash tree has recently been sequenced. Here, we present whole genome DNA methylation data for two *F. excelsior* genotypes with high susceptibility to ADB, and two genotypes with low susceptibility, each clonally replicated. We also include two genotypes of Manchurian ash (*F. mandshurica*), an ash species which has co-evolved with *H. fraxineus* and also has low susceptibility to ADB.

**Results:**

In *F. excelsior,* we find an average methylation level of 76.2% in the CG context, 52.0% in the CHG context, and 13.9% in the CHH context; similar levels to those of tomato. We find higher methylation in transposable elements as opposed to non-mobile elements, and high densities of Non-Differentially Methylation Positions (N-DMPs) in genes with housekeeping functions. Of genes putatively duplicated in whole genome duplication (WGD) events, an average of 25.9% are differentially methylated in at least one cytosine context, potentially indicative of unequal silencing. Variability in methylation patterns exists among clonal replicates, and this is only slightly less than the variability found between different genotypes. Of twenty genes previously found to have expression levels associated with ADB susceptibility, we find only two of these have differential methylation between high and low susceptibility *F. excelsior* trees. In addition, we identify 1683 significant Differentially Methylated Regions (DMRs) (q-value< 0.001) between the high and low susceptibility genotypes of *F. excelsior* trees, of which 665 remain significant when *F. mandshurica* samples are added to the low susceptibility group.

**Conclusions:**

We find a higher frequency of differentially methylated WGD-derived gene duplicates in ash than other plant species previously studied. We also identify a set of genes with differential methylation between genotypes and species with high versus low susceptibility to ADB. This provides valuable foundational data for future work on the role that epigenetics may play in gene dosage compensation and susceptibility to ADB in ash.

**Electronic supplementary material:**

The online version of this article (10.1186/s12864-018-4874-8) contains supplementary material, which is available to authorized users.

## Background

Patterns of genomic DNA methylation vary widely both among [1, 2] and within [3] plant species. Methylation epimutations occur more frequently than genetic mutations in *Arabidopsis* (c. 4.5 × 10^− 4^ epimutations per CG site per generation [4] versus c. 7 × 10^− 9^ base substitutions per generation [5]). An increasing number of plant traits have been found to be under epigenetic control [6], such as fruit ripening in tomato [7] and energy use efficiency in canola [8]. Methylation can play a role in pest or pathogen resistance (for example, demethylation of a promoter region enables expression of a resistance gene in rice [9]) and in response to infection (for example, demethylation in response to infection in rice [10]). Verhoeven et al. [11] found that pathogen and herbivore stress induce varying epigenetic changes in dandelions, meaning that the plants responded to infection in different ways.

Methylation is often involved in silencing duplicated elements in the genome, including duplications among chromosomes, and between maternal or paternal copies of genes [12, 13]. Extensive epigenetic reprogramming after whole genome duplication (WGD) events (e.g. [14–18]), appears to be common: many homeologs retained after WGD events are unequally silenced via DNA methylation or other means (e.g. [19–21]). The vast majority of transposable elements are highly methylated [22, 23] in order to silence their activity; this is considered to be a genome defence mechanism [24, 25].

Methods of DNA sequencing that use chain termination or sequencing by synthesis cannot distinguish between methylated cytosines and unmethylated cytosines with normal DNA preparation methods. Instead, a bisulphite conversion step must be performed before sequencing. This process converts unmethylated cytosines into uracil by deamination, but leaves methylated cytosines intact. Upon PCR amplification of DNA, uracil is converted to thymine. Therefore the overall result from the bisulphite conversion is that unmethylated cytosine is turned into thymine. Genome-wide bisulphite sequencing has been carried out on several crop plants: rice [26, 27], maize [28–30], soybean [19, 31], wheat [32] and tomato [33]. The methylome of *Arabidopsis thaliana* is also well-characterised [3, 4, 22, 34]. To our knowledge, only three tree species have had whole genome bisulphite sequences published; *Populus trichocarpa* (black cottonwood [35]), *Picea abies* (Norway spruce [36]) and *Betula platyphylla* (white birch [37]). Oil palm, *Elaeis guineensis*, has also had whole-genome bisulphite sequencing performed, but described in little detail, by Ong-Abdullah et al. [38]. The methylomes of some trees such as oak [39, 40], have been sequenced using reduced representation bisulphite sequencing (RRBS) which sequences the regions surrounding restriction sites. This technology allows sequencing with deep coverage of many loci, but is not completely genome-wide.

The genome of European ash, *Fraxinus excelsior*, has recently been sequenced [41]. Of 38,852 protein-coding genes annotated in the genome, almost 25,000 appear to be duplicated [41]. Plots of synonymous divergence between duplicates suggest that 2862 derive from a recent WGD event [41], perhaps shared with *Olea europaea* (olive) and therefore common to the Oleaceae family (see also [42]). Another 432 appear to be derived from a less recent WGD, putatively shared with olive and *Mimulus guttatus* which is also in the Lamiales [41]. We hypothesise that many of the homeologs retained in the ash genome since these putative WGD events, may be unequally silenced via differential methylation as a means of gene dosage compensation [43, 44].

European ash tree populations are being severely damaged by ash dieback (ADB), a disease caused by the fungus *Hymenoscyphus fraxineus* [45]. Previous research using associative transcriptomics has identified a number of genes whose expression was significantly associated with ash tree susceptibility to ADB [41, 46], but the method of expression regulation is not yet known. It could be that methylation is involved. We therefore hypothesise that methylation of certain genes could be associated with susceptibility to ash dieback.

Here, we present whole genome bisulphite sequencing data for four genotypes of *F. excelsior* (two genotypes with high, and two with low, susceptibility to ADB) with clonal replicates of each genotype, giving a total of 17 samples. We also sequence two *F. mandshurica* (Manchurian ash) genotypes, one of them clonally replicated, giving a total of three samples (Table 1); this species occurs in the native range of *H. fraxineus* and is reported to have low susceptibility [47, 48]. We describe methylation over various regions of the genome. We investigate the density of Non-Differentially Methylated Positions (N-DMPs, positions consistently unmethylated or completely methylated across all samples) across the genome and associate these with gene models. We investigate levels of methylation in homeolog pairs (gene duplicates retained from putative WGD events) and test for differential methylation within pairs. We investigate the methylation level in ADB susceptibility-associated genes (identified in previous research) to see whether their expression differences could be caused by DNA methylation. We also identify differentially methylated regions (DMRs) between our samples of genotypes with high versus low susceptibility to ADB, both with and without *F. mandshurica* included as a low susceptibility genotype.

**Table 1:** Description and original locations of the ash trees that provided scion material for the grafted trees used in this study

Genotype	Samples	Source location	ADB damage in 2009
*F. mandshurica* (1995–0717 in Hørsholm Arboretum)	F.mand-1	Yasnoya Village, Primorye, Russia (Seed supplied to Hørsholm Arboretum by RBG Kew in 1995)	N/A
*F. mandshurica* (1989–0095 in Hørsholm Arboretum)	F.mand-2, F.mand-3	Dailing, Heilongiang, China (Seed supplied to Hørsholm Arboretum from China in 1989)	N/A
*F. excelsior* Clone 27	F.exc 27–1, F.exc 27–2	Helved, Denmark 55.0094 N, 9.9391 E	90% of samples with > 50% damage
*F. excelsior* Clone 33	F.exc 33–1, F.exc 33–2, F.exc 33–3, F.exc 33–4, F.exc 33–5	Boller, Denmark 55.8343 N, 9.9178 E	70% of samples with < 10% damage
*F. excelsior* Clone 35	F.exc 35–1, F.exc 35–2, F.exc 33–3, F.exc 33–4, F.exc 33–5	Sorø, Denmark 55.3855 N, 11.5851 E	90% of samples with < 10% damage
*F. excelsior* Clone 40	F.exc 40–1, F.exc 40–1, F.exc 40–3, F.exc 40–4, F.exc 40–5	Sorø, Denmark 55.5276 N, 11.7369 E	95% of samples with > 50% damage

Grafts were grown in a common glasshouse environment at University of Copenhagen. The *F. mandshurica* scion materials were from trees germinated from seed in the Hørsholm Arboretum, Copenhagen in 1995 and 1989. The *F. excelsior* genotypes had been tested for ADB susceptibility in clonal field trials established in 1998 and surveyed in 2009 [49]

## Results

### Sequence coverage

We sequenced the methylomes of leaf tissue from twenty trees (Table 1) using whole genome bisulphite sequencing (WGBS). These trees consisted of two grafted replicates of *F. excelsior* Clone 27, and five grafted replicates each of *F. excelsior* Clones 33, 35 and 40, two grafted replicates of one *F. mandshurica* genotype, and one sample of another *F. mandshurica* genotype. Clones 33 and 35 have previously been shown to have consistent low susceptibility to ADB in field trials [49] and *F. mandshurica* is a species with low susceptibility to ADB [47, 48]. After bisulphite conversion and sequencing, a total of 2052 million raw reads were generated, with an average of 102.6 million reads (12× coverage) per sample (Additional file 1).

### Sequence-contexts of methylation

**Fig. 1:**
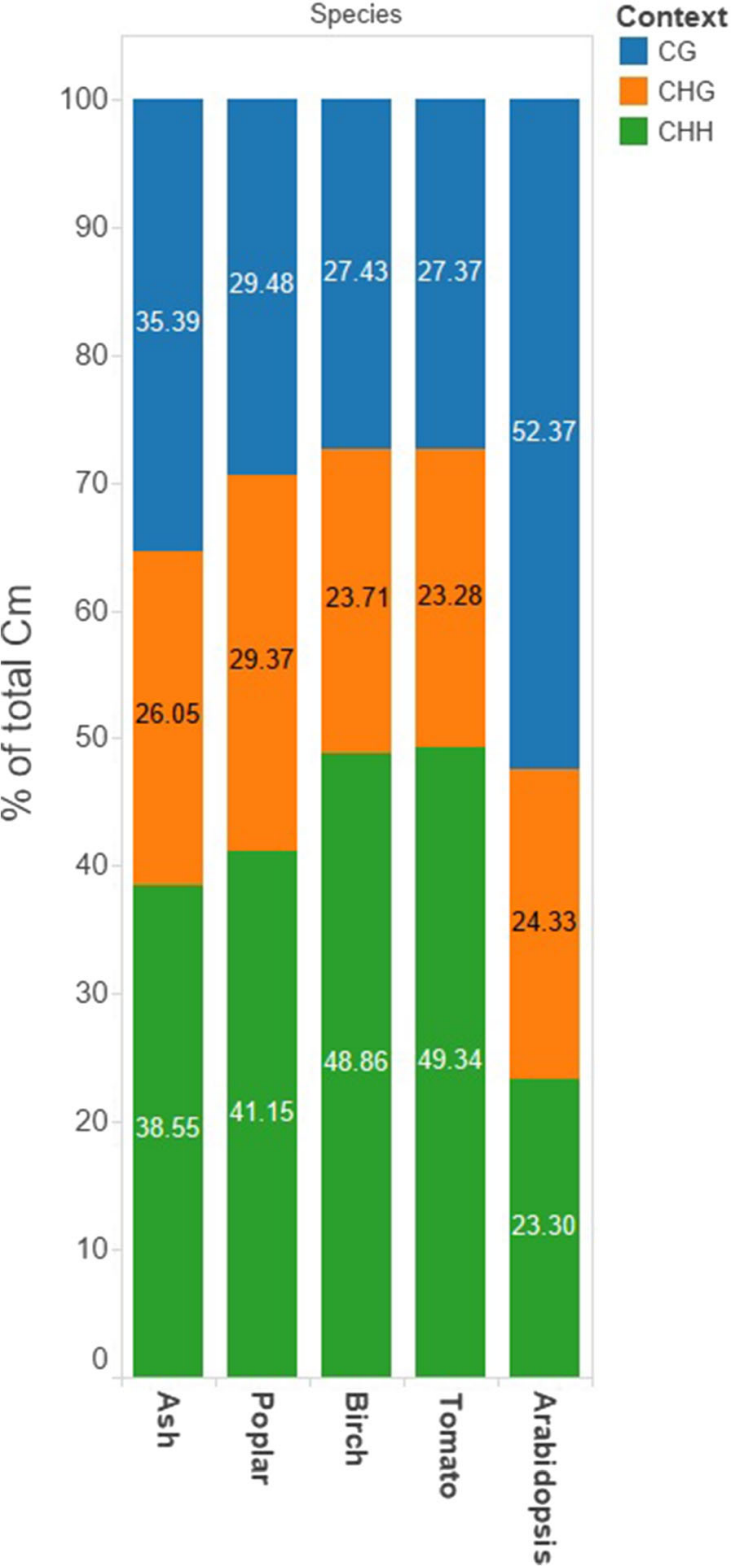
Percentage of methylated cytosines in each sequence context; CG, CHG and CHH (where H = A, C, or T), from pooled mapping of reads from all *F. excelsior* trees. Values taken from ash leaf (this study), *Poplar trichocarpa* (Poplar) leaf [35], *Betula platyphylla* (Birch) vascular tissue [37], *Solanum lycopersicum* (Tomato) leaf [33] and *Arabidopsis thaliana* leaf [33]

Using reads pooled from all *F. excelsior* samples, we gained 125× average coverage of the non-N genome after filtering and mapping. For the 164 million cytosine loci for which we had at least 5× coverage after correction for false positives (see Methods), we found that the average methylation level was 26.6%, further split up into 38.5% for cytosines in the CHH context, 35.4% in the CG context, and 26.1% in the CHG context (Fig. 1).

**Fig. 2:**
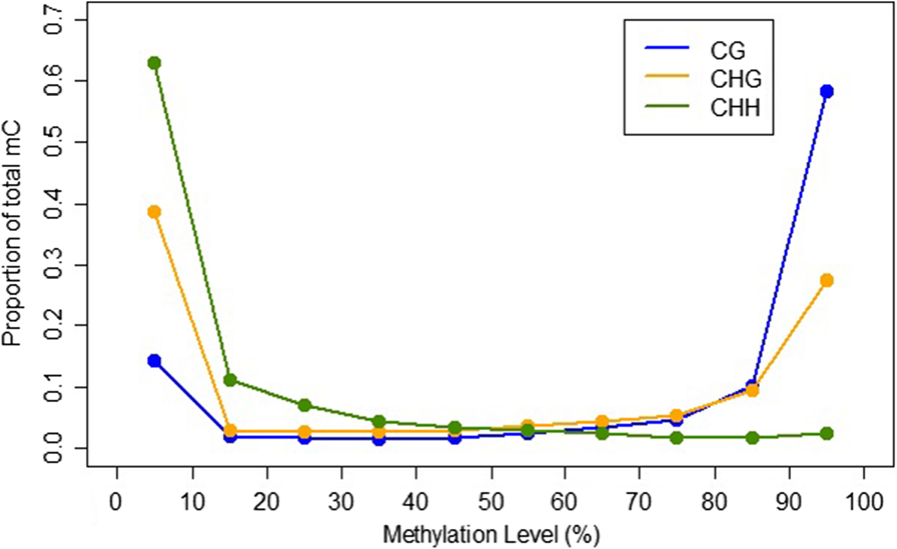
Proportion of methylated bases in each context occurring at various methylation levels in pooled reads from 17 *F. excelsior* trees. Most CHH cytosines are unmethylated or methylated at very low levels, whereas most CG cytosines are methylated at very high levels. Relatively few cytosines are methylated at medium (20–80%) levels

The weighted mean methylation levels [50] across the whole genome were 76.2% for cytosines in the CG context, 52.0% in CHG context and 13.9% in CHH context, which are very similar levels to those found in the tomato methylome [33]. Weighted mean methylation among all 17 *F. excelsior* samples pooled (Fig. 2), showed that cytosines in the CHH and CG contexts tended to have low and high levels of methylation, respectively. Methylation in the CHG context showed a more bimodal distribution, with most cytosines having either very high *or* very low levels of methylation in the pooled reads. Very few cytosines showed an intermediate level of methylation.

**Fig. 3:**
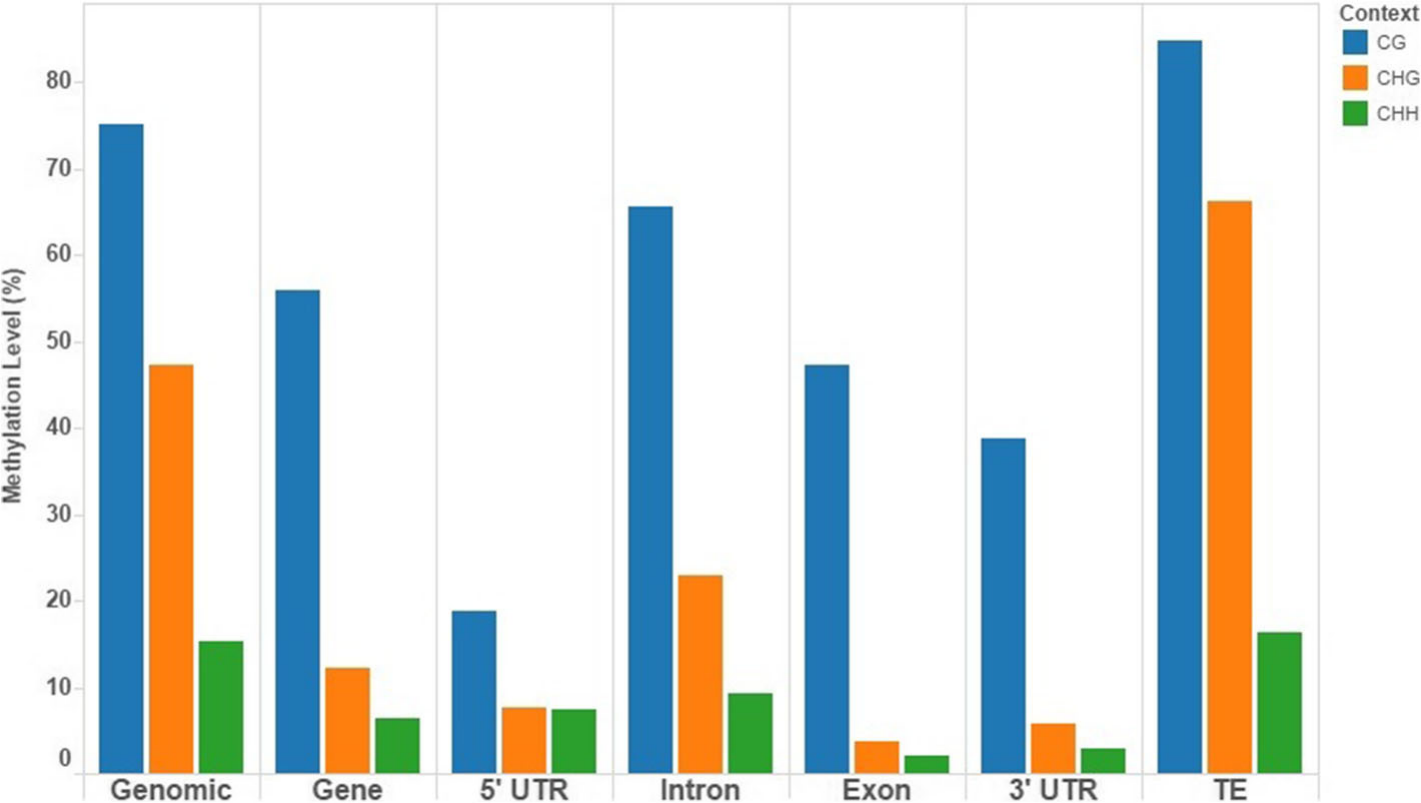
Weighted methylation levels in different genomic elements and contexts for pooled reads from 17 samples of *F. excelsior*

**Fig. 4:**
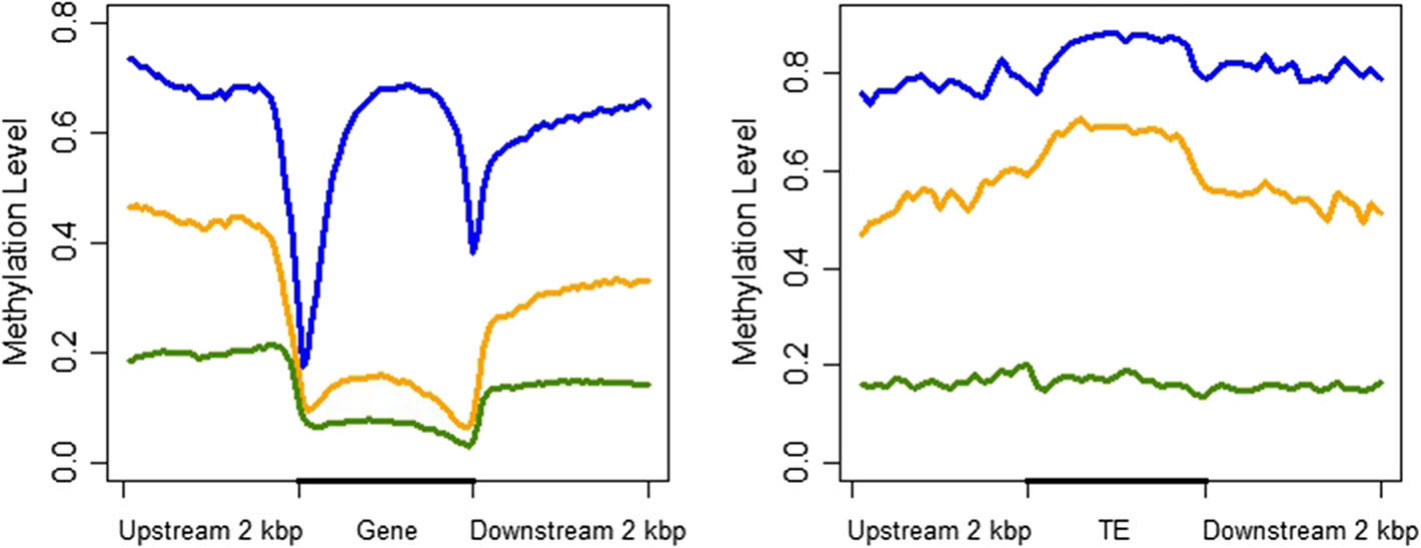
Weighted methylation levels across, and in 2 kbp flanking regions of, genes (left) and transposable elements (TEs, right). Each section of genes and flanking regions was split into 40 bins, and of TEs into 20 bins. Methylation across genes dips at the start and end sites, whereas methylation slightly increases across TEs. Data are from pooled reads from 17 *F. excelsior* individuals

Methylation levels from the pooled samples were lower in gene regions than the genomic average in all sequence contexts, but especially so in the CHG context (Fig. 3). Introns were methylated to a higher degree than exons, and transposable elements (TEs) were substantially more methylated than non-TE genes in all contexts. Sharp dips in methylation level in all contexts were seen at the start and end sites of genes, especially in the CG context, but not in TE genes where methylation slightly increased (Fig. 4). Very similar patterns have been seen in the methylomes of Norway spruce [36], white birch [37] and tomato [33].

Considering each sample separately, 3.1 million cytosines were covered by five or more reads in each of the 17 *F. excelsior* samples. Of these 3.1 million, 459,904 (14.5%) were classed as non-differentially methylated positions (N-DMPs), with 97.5% of these having zero methylation in all samples, and 2.5% being completely methylated in all samples. Of these 459,904 N-DMPs, 23,710 were in the CG context, 21,454 in CHG, and 414,740 in CHH. Sixteen percent (73,724) of these N-DMPs occurred in gene regions; 5456 in CG, 6474 in CHG and 61,794 in CHH contexts. We found variability in the density of N-DMPs occurring within gene regions. Twenty genes with the highest density of N-DMPs, relative to their length, are listed in Table 2, of which all their N-DMPs were completely unmethylated. The majority of these genes hold functions that are very conserved across plants and/or eukaryotes, such as those associated with photosynthesis (e.g. NADH hydrogenase subunits), or various ribosomal proteins.

**Table 2:** Twenty genes with highest density of N-DMPs

Gene ID	N-DMP Density	Functional Annotation
376950	0.2321	None
016370	0.2119	Pg1 protein, lyase activity
376920	0.1712	NADH dehydrogenase subunit 2
016400	0.1689	NADH dehydrogenase subunit 2
376910	0.1668	NADH dehydrogenase subunit 2
009390	0.1602	ATP-ase, AAA-type
016410	0.1558	NADH dehydrogenase subunit 2
238880	0.1545	Ribosomal protein L5
285710	0.1488	Ribosomal protein L2
078310	0.1468	Photosystem II CP43 Chlorophyll partial
196460	0.1455	Ribosomal protein S12
016420	0.1454	ATP-ase, AAA-type
016390	0.1432	Ribosomal protein S7
277070	0.1429	NADH dehydrogenase subunit 2
376900	0.1424	ATP-ase, AAA-type
363290	0.1418	Ribosomal protein L23
197880	0.1409	NADH dehydrogenase subunit 4
137570	0.1405	Photosystem I p700 apoprotein a1
190110	0.1403	NADH dehydrogenase subunit 2
221320	0.1396	Photosystem I p700 apoprotein a2

All N-DMPs were completely unmethylated in all *F. excelsior* samples. Density calculated as: #N-DMPs/(gene length*2) (where multiplication by two takes into account both strands of DNA)

### Differential methylation of homeologs

**Fig. 5:**
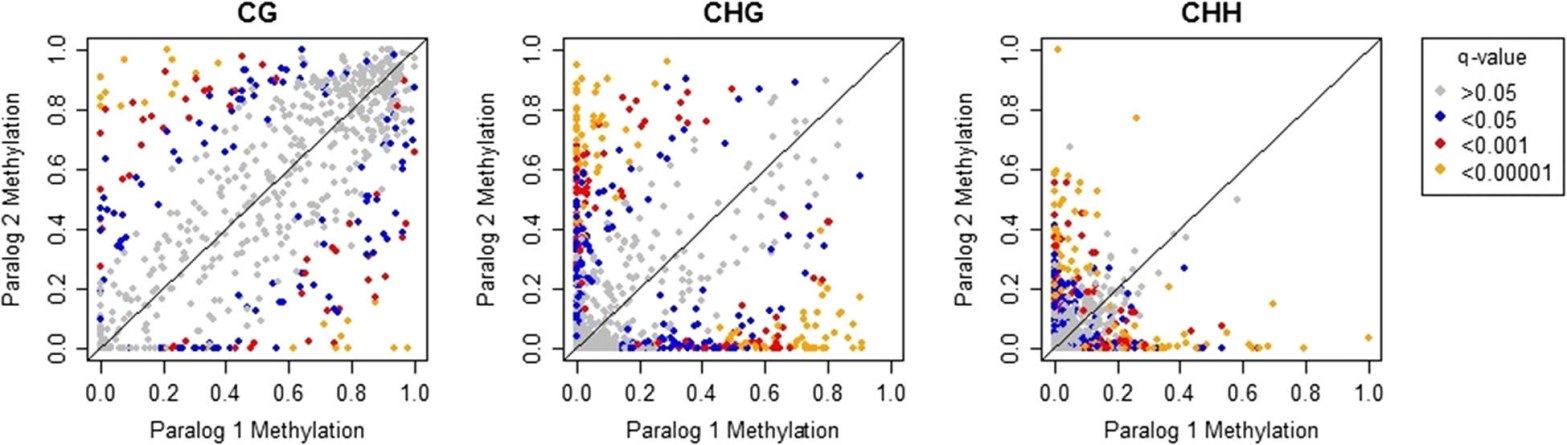
Differential methylation in homeologs of sample F.exc 33–2 (as an example), split into CG, CHG and CHH contexts, plotted as methylation of one paralog in a pair against methylation of the other. Q-values shown are FDR-adjusted *p*-values, to correct for multiple tests. Low q-values tend to occur in top-left and bottom-right corners of the graphs (where one paralog has low methylation and the other high) and along each zero axes; where one paralog has zero methylation and the other has at least a medium level

By extracting the 3297 pairs of genes putatively involved in two WGD events (2862 from the lower Ks values peak, and 432 from the higher Ks values peak) and measuring their methylation, we were able to investigate differential methylation between homeolog pairs. As an example, the methylation values of homeologs in one sample (F.exc 33–2) and adjusted q-values from a linear model, are shown in Fig. 5. The total number of homeologs identified as differentially methylated in each sample depended on the overall read coverage for each sample. Table 3 shows the results for each tree. On average, 23.4% of the homeolog pairs were differentially methylated in the CG context in each sample, with 57/1066 pairs consistently differentially methylated in at least ten samples. In the CHG context, 28.6% of pairs were differentially methylated, 239/1106 across ten samples or more, and in the CHH context, 24.5% of pairs were differentially methylated, 396/1814 across ten samples or more. These values were much higher than other studied plant species, for example Schmitz et al. [19] found 602/9793 (6.1%) homeolog pairs in soybean were differentially methylated, and in *Arabidopsis thaliana* (where they re-analyzed data from [4, 51]) they estimated that 4/497 (0.8%) of homeologs were differentially methylated. When we analysed homeologs from the two Ks peaks in the ash genome separately we found greater differentiation among homeologs in the higher Ks values peak than in the lower Ks values peak in all cytosine contexts (Kolmogorov–Smirnov tests, *p*-values of 0.0017, <2e-16, 2.2e-16 for CG, CHG and CHH contexts, respectively). Homeolog pairs with the lowest *p*-values from the test for differential methylation across multiple *F. excelsior* samples are shown in Additional file 2. These gene pairs all had a consistent direction of differential methylation i.e., the gene with the lower methylation value was the same across all samples. Gene Ontology (GO) enrichment in the set of differentially methylated homeologs was performed using the entire set of *F. excelsior* homeologs as a reference; the results are shown in Additional file 3, with different tabs for Biological Process (BP), Cellular Component (CC) and Molecular Function (MF) GO terms. Some of the most significantly enriched GO terms in the BP category were those involved in cell growth and cell tip growth (e.g. GO:0048588 and GO:0009932), in the CC category many were expressed in the chloroplast / plastid (e.g. GO:0009536, GO:0009532, and GO:0009941), and in the MF category many were involved in binding activities such as anion binding (GO:0043168), purine ribonucleoside binding (GO:0032550), and adenyl ribonucleotide binding (GO:0032559).

**Table 3:** Percentage of homeolog pairs (with at least ten cytosines covered by > 3 reads) that are significantly differentially methylated for each *F. excelsior* tree

Sample	Significantly differentially methylated gene WGD pairs out of total with sufficient coverage		
	CG	CHG	CHH
F. exc 27–1	128 / 553 (23%)	314 / 1045 (30%)	516 / 2281 (23%)
F. exc 27–2	186 / 700 (27%)	377 / 1263 (30%)	624 / 2422 (26%)
F. exc 33–1	131 / 475 (28%)	251 / 893 (28%)	472 / 2163(22%)
F. exc 33–2	465 / 1690 (28%)	622 / 2214 (28%)	808 / 2992 (27%)
F. exc 33–3	304 / 1189 (26%)	485 / 1823 (27%)	768 / 2836 (27%)
F. exc 33–4	300 / 1094 (27%)	437 / 1688 (26%)	694 / 2726 (26%)
F. exc 33–5	50 / 177 (28%)	139 / 410 (34%)	371 / 1577 (24%)
F. exc 35–1	212 / 846 (25%)	403 / 1472 (27%)	655 / 2599 (25%)
F. exc 35–2	296 / 1095 (27%)	462 / 1689 (27%)	738 / 2722 (27%)
F. exc 35–3	256 / 1006 (25%)	430 / 1650 (26%)	654 / 2687 (24%)
F. exc 35–4	110 / 488 (23%)	259 / 933 (28%)	536 / 2240 (24%)
F. exc 35–5	15 / 75 (20%)	71 / 175 (41%)	210 / 930 (23%)
F. exc 40–1	7 / 37 (19%)	28 / 89 (32%)	133 / 672 (20%)
F. exc 40–2	279 / 1070 (26%)	486 / 1709 (18%)	719 / 2745 (26%)
F. exc 40–3	254 / 1032 (25%)	447 / 1671 (27%)	629 / 2747 (23%)
F. exc 40–4	250 / 1028 (24%)	444 / 1622 (27%)	647 / 2699 (24%)
F. exc 40–5	175 / 666 (26%)	367 / 1203 (31%)	648 / 2440 (27%)

Samples already identified as low coverage (F.exc 40–1, F.exc 35–5, F.exc 33–5) have lower numbers of homeolog pairs meeting the coverage criteria, therefore percentages for these trees may be skewed by low sample size

### Differences among samples

**Fig. 6:**
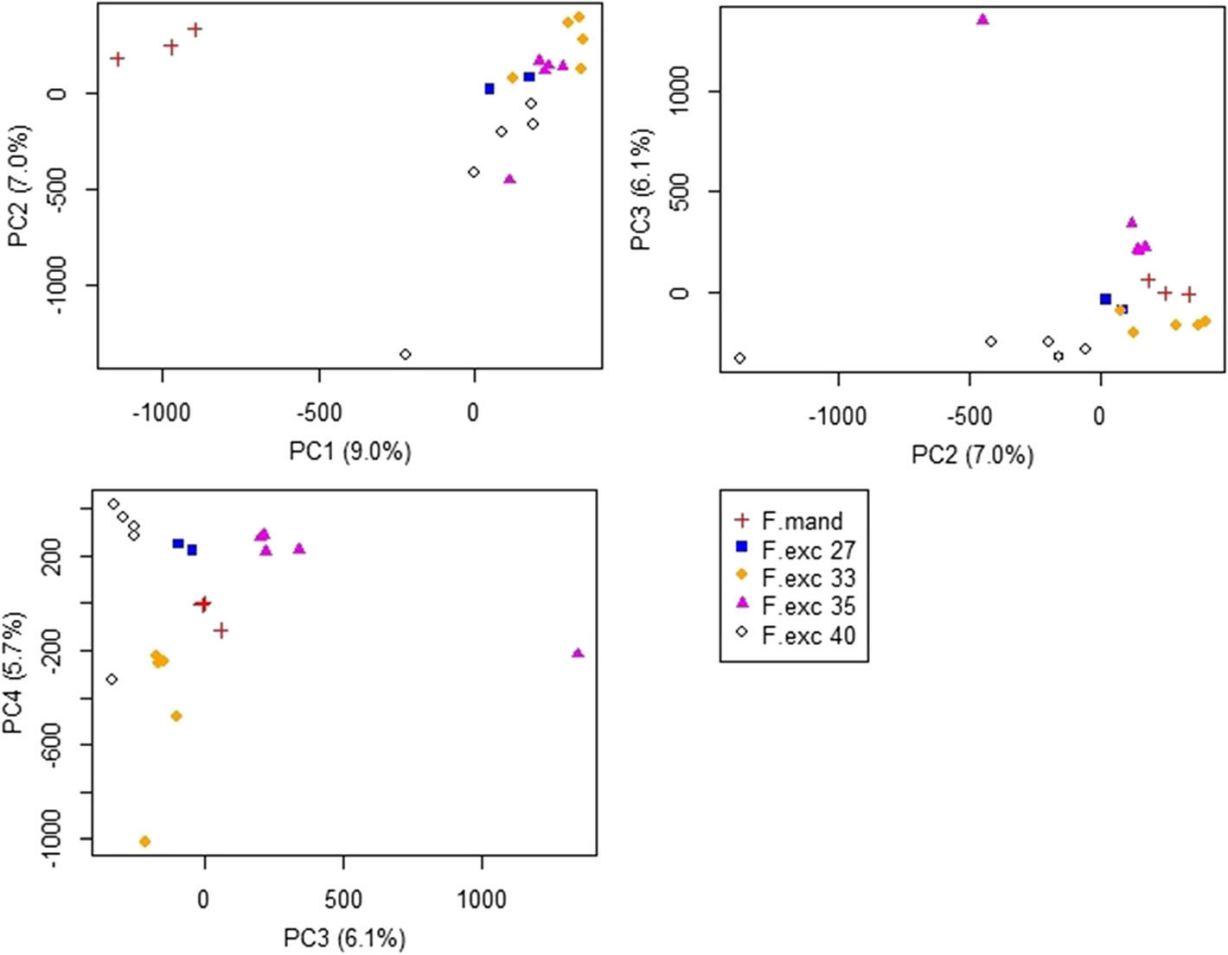
Principal Components Analysis of methylation values from 400,000 cytosines across all samples. *F. mandshurica* trees are split from *F. excelsior* trees along PC1. Three outliers represent low coverage samples: pink triangle outlier on PC3 is F.exc 35–5, black diamond outlier on PC2 is F.exc 40–1, and orange diamond outlier on PC4 is F.exc 33–5. SNP positions with highest loadings along PC1 are listed in Additional file 4

Using Principal Components Analysis (PCA), we were able to distinguish samples from the two *Fraxinus* species based on their methylation patterns (Fig. 6). The plot of PC1 vs PC2 clearly showed separation of the *F. mandshurica* trees (red crosses) away from all *F. excelsior* individuals along the PC1 axis, but the *F. excelsior* trees were not separated from each other very much. Using the loadings of each cytosine position for PC1, we obtained a list of the top 40 positions responsible for most of the separation between the two species (Additional file 4). The vast majority of these positions were not within gene regions, with the exception of two: Contig2376 position 21,815 was within gene 119,700, a phospholipid-translocating ATPase, and Contig 8085 position 87,294 lay within gene 356,660, an RNA recognition motif-containing protein.

**Fig. 7:**
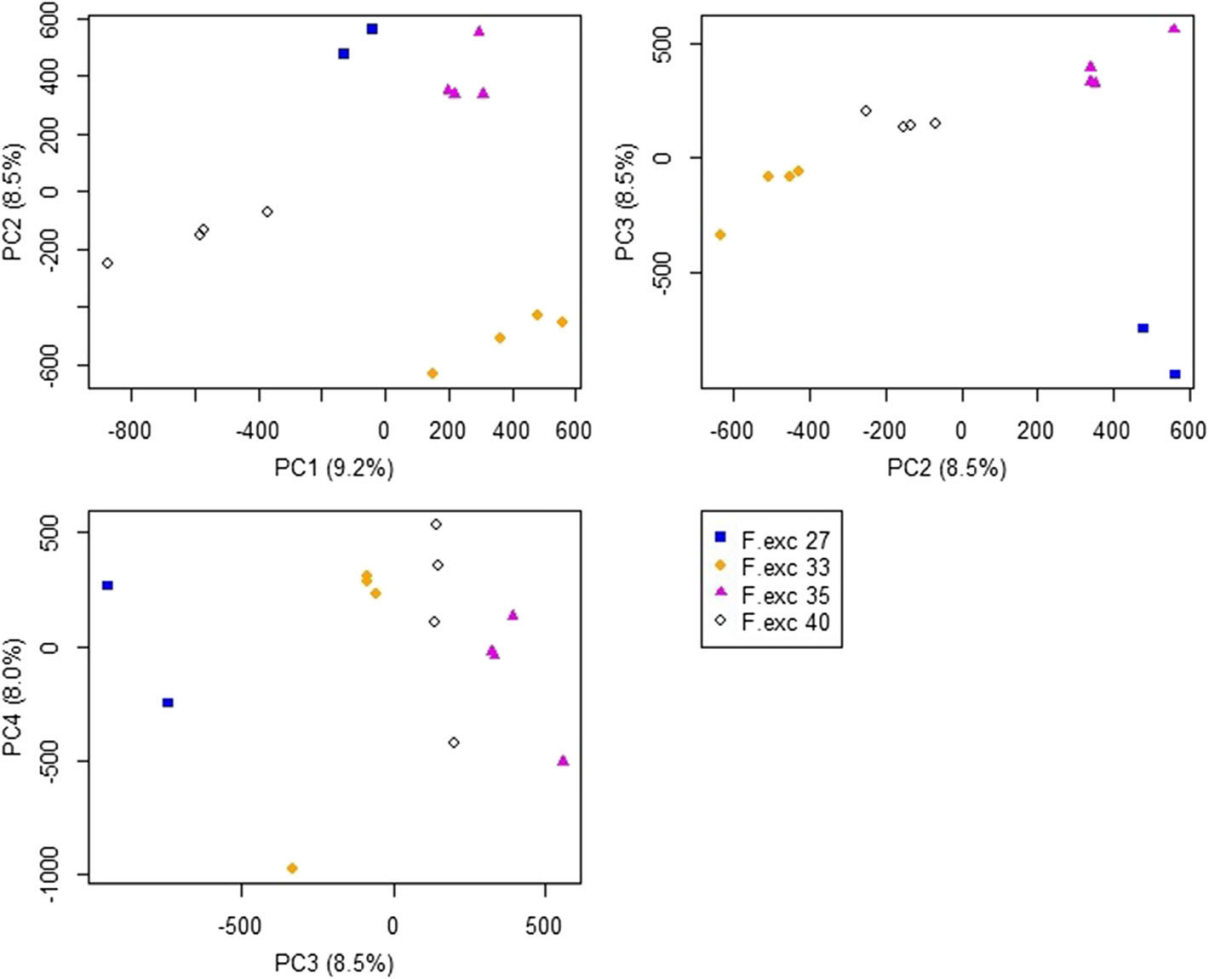
Principal Components Analysis of methylation values from 400,000 cytosines for high coverage *F. excelsior* samples. F.exc 33–5, F.exc 35–5, and F.exc 40–1 were removed from the PCA, leaving samples that cluster highly within each genotype. However some variation in methylation is visible between samples within each genotype

There were three outliers on the PC plots which were all very low coverage *F. excelsior* samples. In Fig. 6 the pink triangle outlier on PC3 is F.exc 35–5, the black diamond outlier on PC2 is F.exc 40–1, and the orange diamond outlier on PC4 is F.exc 33–5. All of these samples had an average genome coverage after mapping of < 5×. When these three low-coverage samples were excluded from the analysis, the four *F. excelsior* genotypes became distinguishable from each other along the main PCs (Fig. 7). This analysis demonstrates the need for sufficient genome coverage, ideally > 10× raw read coverage, and > 5× after QC and mapping, before attempting to compare methylomes.

**Fig. 8:**
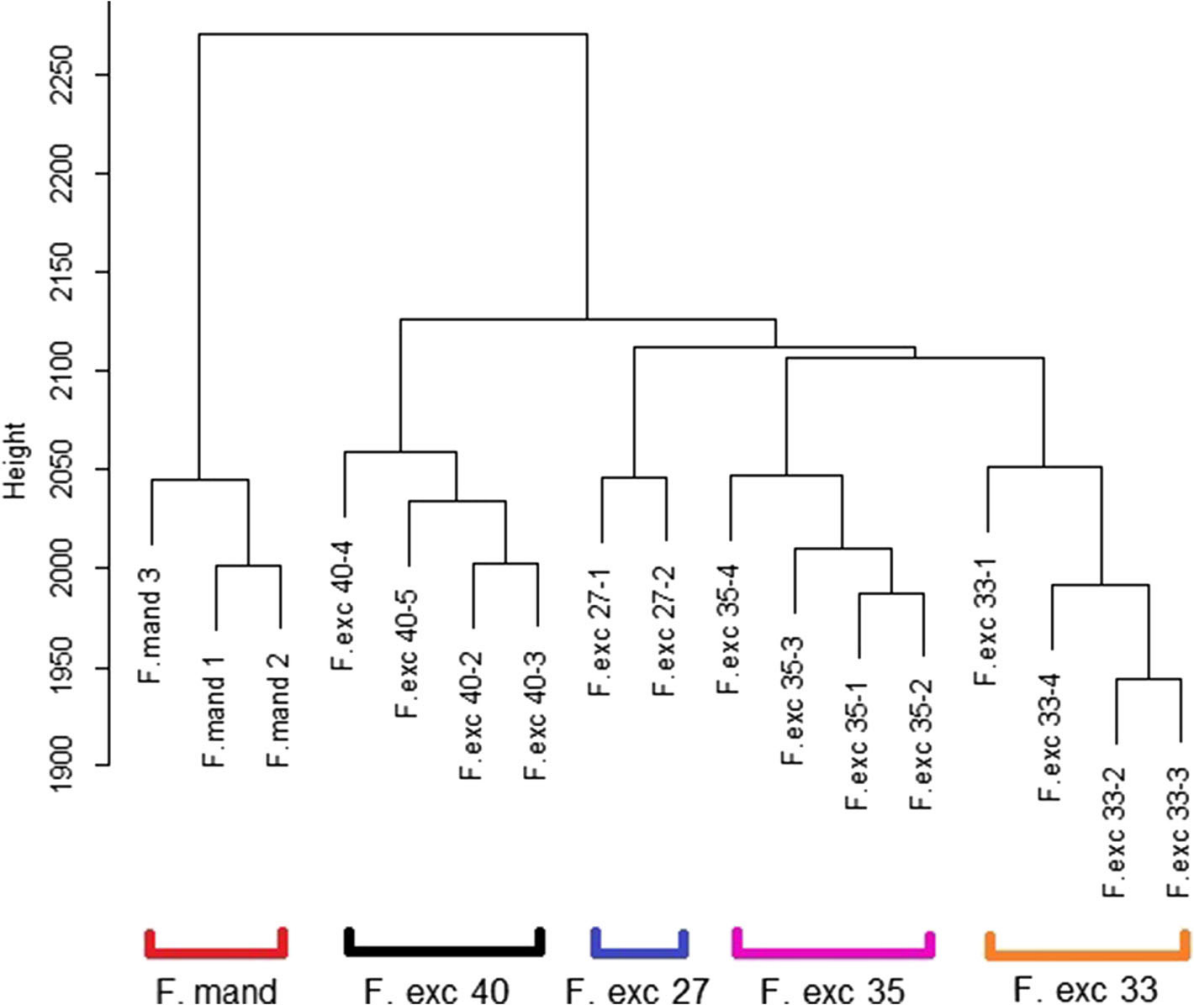
Hierarchical clustering of high coverage samples using methylation values from 400,000 positions, shows that samples cluster within each genotype. All samples shown have average genome coverage > 5×, therefore F.exc 35–5, F.exc 40–1, and F. exc 33–5 are removed. These outlier samples clustered away from all other *F. excelsior* genotypes when included, as if they were outgroups

The PCA with low coverage samples excluded gave evidence for genotype-specific methylation patterns, as samples from the same genotype clustered together. Some variation remained between the clones within each genotype. Hierarchical clustering (Fig. 8) showed the three *F. mandshurica* trees to form an outgroup by themselves away from the other *F. excelsior* samples. Without the three low coverage samples described previously, the *F. excelsior* samples grouped clearly into their genotypes, giving a similar pattern to the PCA plot.

**Fig. 9:**
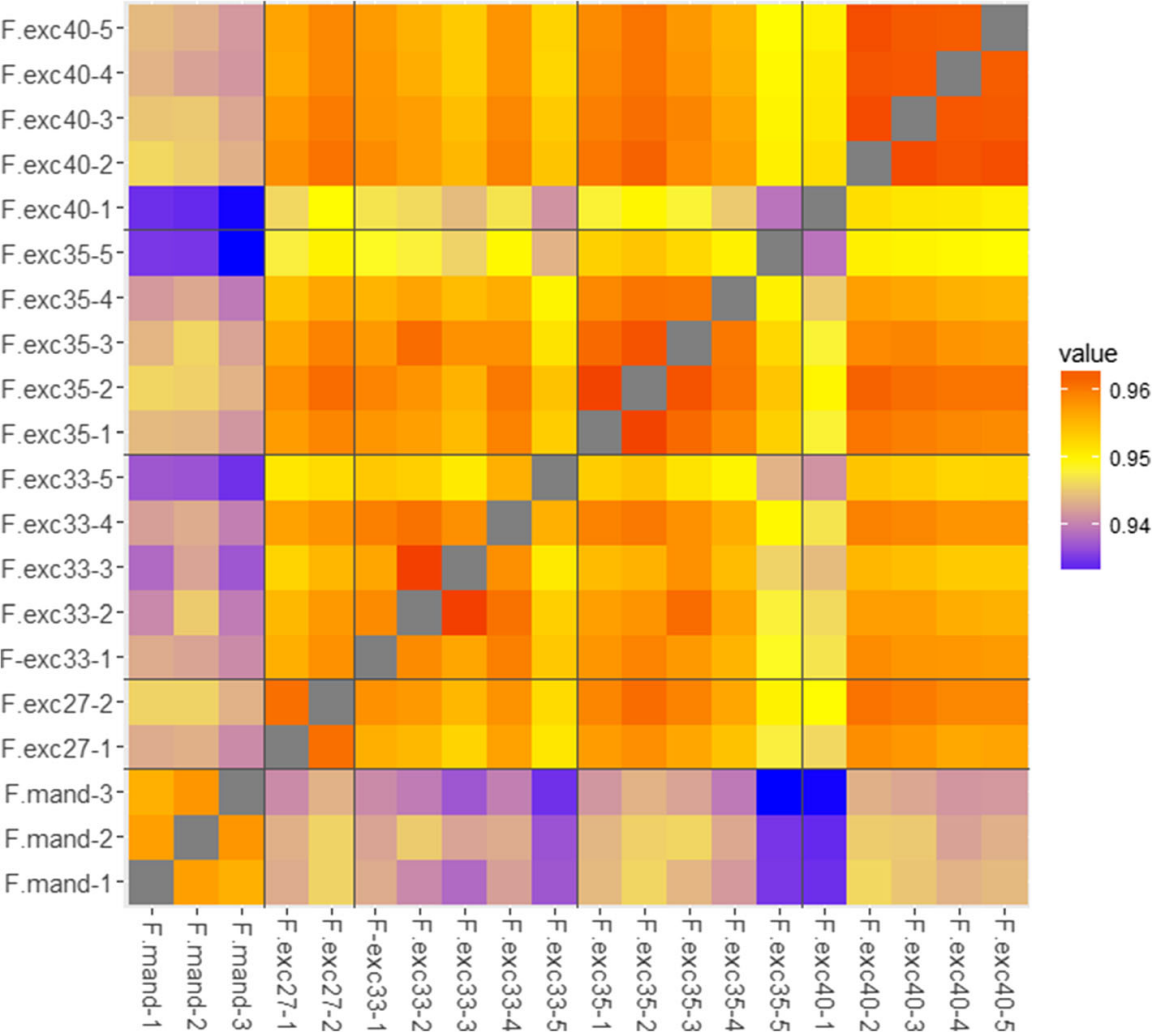
Correlation matrix heatmap of all samples using Pearson’s Correlation coefficient of methylation values. All samples show very slightly higher correlation with samples of the same genotype (~ 0.96), than with samples of a different genotype(~ 0.955), reflecting possible genotype-specific methylation patterns but still showing variation between clones within each genotype. *F. mandshurica* methylomes show low correlation with all *F. excelsior* methylomes (~ 0.94). The low coverage *F. excelsior* samples, particularly F.exc 35–5, F. exc 40–1, and to some extent F.exc 33–5, also show lower correlation with other *F. excelsior* trees (~ 0.95)

To investigate the difference in variation between samples within a genotype compared to between genotypes, we performed a Pearson’s correlation test of methylation values for every pairwise combination of samples. A heatmap of the correlation matrix is shown in Fig. 9. Again, it can be clearly seen that the *F. mandshurica* individuals had lower correlation values with all other *F. excelsior* trees (~ 0.94). The low coverage samples (F.exc 33–5, F.exc 35–5 and F. exc 40–1) also showed slightly lower correlation values to other *F. excelsior* genotypes (~ 0.95), and their difference was in line with their average coverage values (e.g. F.exc 40–1 had the lowest coverage at 2.8×, and also had the lowest correlation values to other samples). Correlation values within genotypes (~ 0.96) were higher than between genotypes (~ 0.955), but clearly not by a large amount.

### Differential methylation associated with ash dieback susceptibility

**Fig. 10:**
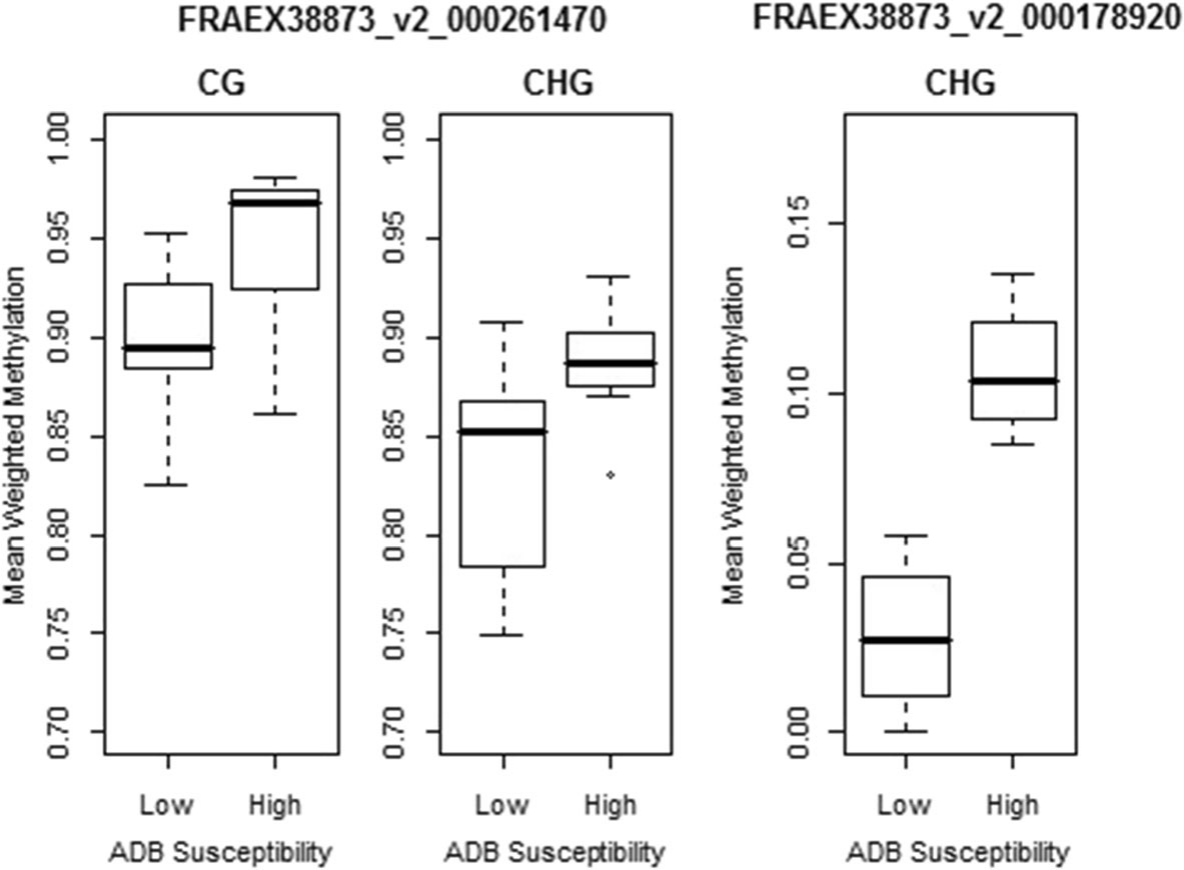
Boxplots of mean weighted methylation levels for high and low susceptibility samples, for two genes found to be significantly associated with ADB susceptibility [41]

We also looked for differences in methylation patterns between trees with low and high ADB susceptibility. Firstly, we investigated methylation patterns in twenty genes that were previously found to have expression levels associated with ADB susceptibility [41]. Only two of these genes were significantly differentially methylated between the two groups; 261470 (soc1-like protein) was significant both in the CG and CHG context, and 178920 (cinnamoyl-CoA reductase 2) was significant in the CHG context (Fig. 10). All had higher methylation in the high susceptibility group. However, after adjusting *p*-values for multiple tests, only 178920 remained significant. Detailed results are shown in Additional file 5.

We used the program metilene v0.2–6 [52] to search for regions in the genome that are differentially methylated between the high and low susceptibility *F. excelsior* samples. In total, 1683 significant DMRs were found between the low and high susceptibility *F. excelsior* samples at q < 0.001, with 103 in the CG context, 112 in CHG and 1468 in CHH (Additional file 6). Of these DMRs, 20.2% overlapped with genes, however there were stark differences in the level of gene association between the three sequence contexts: 41/103 (39.8%) CG-DMRs overlapped with a gene, compared to 70/112 (62.5%) CHG-DMRs and 169/1168 (14.5%) CHH-DMRs. Of gene regions overlapping these DMRs, three were present in both CG- and CHG-DMRs, meaning that they could be consistently differentially methylated between low and high susceptibility trees in both CG and CHG contexts. These genes were 043910 (DMR co-ordinates Contig1417: 73501–73,781 in CG and Contig1417: 73461–73,861 in CHG), which had no annotation, 137200, a mechanosensitive ion channel domain-containing protein, *MscS* (DMR co-ordinates Contig2609: 64538–64,958 in CG and Contig2609: 64,573–64,977 in CHG), and 197890 (DMR co-ordinates Contig3629: 24,849–25,184 in CG and Contig3629: 24,832–25,193 in CHG), also with no annotation. None of the twenty genes previously identified [41] as having expression levels associated with ADB susceptibility had significant DMRs in any sequence context in this analysis.

When we added the *F. mandshurica* samples into the DMR comparison, we found that 1018 of the DMRs previously identified were no longer significant, and only 665 remained significant at q < 0.001 (40 in CG context, 30 in CHG, and 595 in CHH, Additional file 7). Out of these 665 DMRs remaining, 352 had become more significant (smaller q-value) after adding in the *F. mandshurica* samples (Additional file 8), and 313 had become less significant (larger q-value, but still < 0.001). One such DMR (Contig2999:147,267–147,483) overlapped with a gene (gene number 162300) encoding a glycosyl transferase enzyme.

## Discussion

The overall level of cytosine methylation in the leaf methylome of *Fraxinus excelsior* is similar to leaves of *Populus trichocarpa* [35], but less so to vascular tissue of *Betula platyphylla* [37] and leaves of tomato [33], despite the fact that tomato is a fellow member of the Asterid clade with ash. However, the weighted average methylation levels (76.2% in the CG context, 52.0% in CHG and 13.9% in CHH) are very similar to those calculated in the tomato methylome [33]. We also find that methylation is increased in transposable elements in comparison to non-mobile genes, showing patterns similar to that of white birch [37], and Norway spruce [36]. We find that completely unmethylated cytosines are enriched in housekeeping type genes that require constant levels of gene expression, such as those involved in photosynthesis pathways.

Homeologous gene pairs putatively retained from WGD events are found to show more frequent differential methylation in ash than was found by a similar study in soybean and *Arabidopsis* [19]*.* One difference between the findings in soybean and ash is that in the CG context, methylation of homeologs in soybean were mostly equal, leading the authors to not consider CG methylation in their analysis of differential methylation. CG methylation has been found to not be as repressive as CHG and CHH methylation; Wang et al. [21] studied methylation in cassava paralogs, and found that the gene with higher CG-methylation often had higher expression than its paralog. In contrast to soybean [19], we find that a large number of homeologs in ash are differentially methylated in the CG context. It should also be noted that the methods for identifying differentially methylated homeologs in soybean [19], were quite different to our linear modelling approach as the authors selected any homeolog pair where one had < 0.5% methylation and the other > 2.5%. We examine homeolog pairs from two separate putative WGD events in ash, and find that apparently older retained homeolog pairs seem to have higher levels of differential methylation than younger homeologs. Among some of the most significant GO terms enriched in differentially methylated homeologs are those with binding activities (e.g. anion binding, nucleotide binding), cell growth and those expressed in the chloroplast / plastid.

We find that methylation patterns between the genotypes of *F. excelsior* are more highly correlated than between genotypes of *F. excelsior* and *F. mandshurica* in homologous areas of the genome. We also identify positions in these regions that show very different levels of methylation between the genotypes of the two species, and are responsible for much of their separation in PCA. Although differences could be caused by mapping *F. mandshurica* reads against a *F. excelsior* genome which would introduce mismatches and reduce mapping quality, the steps taken to correct for coverage (we only used sites sufficiently covered in all samples) and for mismatches, lead us to believe that these potential confounding issues have a negligible effect on the results. Indeed, the *F. excelsior* samples with lower average read coverage than any of the *F. mandshurica* samples, are still clustered nearer to the other high coverage *F. excelsior* samples than those of *F. mandshurica* are.

As the cloned replicates cluster into their genotype groups, we also conclude there are genotype-specific methylation patterns. These could lead to phenotypic differences between genotypes. There is also epigenetic variation between the clones within each genotype, which thus cannot be attributed to genetic differences. This could represent epigenetic stochasticity in the trees, or subtle variation within the controlled environment. Although all trees were grown in a common environment, it should also be noted that the clones were made by grafting scion materials from the same tree onto rootstocks. Some epigenetic differences between clones may have been present while they were still separate branches of the same tree. Other changes could have occurred during or after the grafting process. Low read coverage skews methylation values enough so that poorly covered samples appear as outliers from other trees of the same genotype. We therefore suggest that further whole-genome methylation studies require an average genome coverage of at least 5X after quality trimming and filtering.

Out of twenty genes previously found to have expression levels associated with ADB susceptibility [41], we find one of these has highly significant methylation level differences between the high and low susceptibility trees. In the previous study, the expression levels of gene 178920 were shown to be higher in high susceptibility trees. This doesn’t seem to fit with the higher CHG methylation levels observed in this gene in the present study, as CHG methylation is usually correlated with suppressed gene expression. The enzyme cinnamoyl-CoA reductase 2 encoded by gene 178920 is part of a phenylpropanoid biosynthesis pathway. Phenylpropanoids form parts of structural compounds which, among other functions, provide defence against fungal pathogens and herbivores [53, 54].

We test for the presence of DMRs between the high and low susceptibility trees, where a total of 1683 were found. One gene associated with some of the most significant DMRs is the mechanosensitive ion channel domain-containing protein, *MscS.* *MscS* proteins respond when the cell is in osmotic stress through mechanical tension changes in the membrane [55]. ADB is thought to cause osmotic stress in cells by causing necrosis in, among other tissues, the xylem vessels [56]. We speculatively hypothesise that the low susceptibility trees (which have an extremely hypomethylated 137200 gene compared to the high susceptibility samples), could have increased expression of this *MscS* protein, and can therefore cope better with the osmotic stress they are put under during ADB infection. One DMR that becomes more significant when *F. mandshurica* samples are included in the analysis, overlaps with a glycosyl transferase gene (162300). These enzymes catalyze the formation of glycosidic bonds in the formation of glycosides. Previous research has found that levels of iridoid glycosides are associated with ADB susceptibility [41], and therefore the differential methylation of a glycosyl transferase enzyme could contribute to this association. We therefore suggest these loci as well as other DMRs identified, as candidates for further study.

## Conclusion

Our results fit well with previous studies of DNA methylation in plants, in terms of patterns of methylation in the genome. Once we have excluded samples with low read coverage (an average of < 5× across the whole genome), we show expected patterns of differentiation within and among species. This suggests that we have a reliable data set. We find a higher frequency of differentially methylated homeolog pairs in ash than other plant species previously studied. We also find of a set of genes with differential methylation between genotypes and species with high versus low susceptibility to ADB. This provides valuable foundational data for future work on the role that epigenetics may play in gene dosage compensation and susceptibility to ADB in ash.

## Methods

### Samples

We were given leaf samples of European and Manchurian ash tree grafts grown in a common greenhouse environment at the University of Copenhagen. This study used twenty samples, consisting of: three samples of two *F. mandshurica* genotypes grafted from trees in the Hørsholm Arboretum; two samples of *F. excelsior* genotype “clone 27”; and five samples each of *F. excelsior* genotypes “clones 33”, “clone 35” and “clone 40”. The original native forest locations of these genotypes are shown in Table 1. The *F. excelsior* genotypes had previously been tested in clonal field trials [49], using natural inoculation by the *Hymenoscyphus fraxineus* fungus. Susceptibility was measured in these trials using crown damage over replicates for each genotype. Clones 27 and 40 were among the most susceptible, with approximately 95% showing greater than 50% crown damage. Clones 33 and 35 were the least susceptible, with 70 and 90% of samples, respectively, showing less than 10% crown damage [49].

### DNA extraction and sequencing

DNA was extracted from dried leaves of the twenty samples in December 2013 at the University of Copenhagen, using Qiagen DNeasy Plant Mini Kit (Qiagen, Hilden, Germany) and checked for quality and quantity using Nanodrop (Thermo Scientific, Waltham, MA, USA). DNA samples were sent to the Genome Centre at QMUL where they were quantified using Qubit (Invitrogen Carlsbad, CA, United States) and vacuum concentrated. Between 200 ng and 500 ng of DNA from each sample were bisulphite-converted at the Genome Centre using the EZ DNA Methylation Gold kit (Zymo Research, Irvine, CA, USA). Libraries were prepared using EpiGnome Methyl-seq kit (Epicentre, Madison, WI, USA), and checked on an Agilent Tape Station using the DNA1000 High Sensitivity Screen Tape assay (Agilent, Santa Clara, CA, USA) and sequencing was carried out on an Illumina HiSeq 2500 instrument (Illumina, San Diego, USA), using 2 × 100 bp paired reads.

### Data analysis, QC and filtering

Raw reads were imported into the CLC Genomics Workbench v8 (Qiagen, Aarhus, Denmark) where QC steps were performed (minimum Phred score of 20, all ‘N’ nucleotides removed, minimum read length of 50 bp and sequencing adapters removed). Trimmed reads were exported from the CLC Genomics Workbench and mapped to the BATG-0.5 ash reference genome [41] using BSMAP v2.90 [57] with the following parameters changed from default: “-3” (uses three nucleotide mapping approach), “-w 20” (maximum number of equal best hits to count), and “-g 3” (maximum size of gaps). Using the methratio.py python script included in the BSMAP package, duplicate reads were removed and methylation levels were calculated for all cytosines with strand coverage of at least four reads. Methylation levels were calculated using: C_m_ / (C_m_ + C_u_) where C_m_ is the number of reads supporting a methylated cytosine, and C_u_ equals the number of reads supporting an unmethylated cytosine. False positive methylation levels obtained from the unmethylated chloroplast genome were used to calculate the efficiency of bisulphite conversion for each sample. From all the chloroplast cytosines examined, the conversion efficiency (%) was calculated as 100*(1-(C_m_ / (C_m_ + C_u_))). To exclude false positive positions caused by incomplete conversion, all nuclear positions that did not have zero methylated coverage were each tested for significance using a binomial test [34]. The false positive rate (inverse of the conversion efficiency) for each sample was used as the expected probability in a binomial test for every cytosine in that sample, with *p*-values then corrected for multiple tests using the Benjamini-Hochberg method of the ‘p.adjust’ function in R. Where cytosines had FDR > 0.05 (i.e. not significantly different from the false positive rate), the number of methylated reads supporting these positions was then set to zero, to remove any reads that likely show false positive methylation due to incomplete bisulphite conversion [50]. Cytosines at known C- > T or G- > A SNP loci were filtered out of all files, using 5.1 million polymorphic positions obtained from the range-wide diversity panel described in Sollars et al. [41]. The genome annotation file ‘Fraxinus_excelsior_38873_TGAC_v2.gff3’ (available at ashgenome.org) was used to define gene, intron, exon and UTR regions, and the file ‘Fraxinus_excelsior_38873_TGAC_v2.possible_transposable_elements.txt’ was used to define which genes were transposable elements. To calculate averages across regions, we used weighted methylation levels instead of mean methylation [50]. Weighted mean methylation levels adjust the weight that each positions gives to the average calculation based on its coverage and use the following calculation: *ΣC*_*m*_/*Σ*(*C*_*m*_ + *C*_*u*_) [50].

### Differential methylation between homeologs

Evidence for two WGD events in the ash genome was found in Sollars et al. [41], using alignments of homeologs and calculating Ks (synonymous substitutions per synonymous site). Pairs of homeologs were extracted from the complete list of paralogs based on their Ks value (2862 with Ks values between 0.2 and 0.4, and 432 with Ks values between 0.5 and 0.8), to ensure that only those derived from the WGD events were considered. Using logit-transformed mean methylation levels across these homeologs, we investigated differential methylation between the gene pairs using linear modelling (R function ‘lm’) to test for significant differences. Only genes that contained at least ten cytosines covered by > 3 reads were used in this test. The logit transformation was used in order to correct for data limits being zero and one, so that a difference between 0.9 and 1.0 is equal to a difference between 0 and 0.1. Values of 0 and 1 were adjusted by 0.001 in order to prevent their logit-transformed values being infinity. All *p*-values resulting from the multiple linear model tests were then corrected for multiple tests using the Benjamini-Hochberg method of the ‘p.adjust’ function in R. The linear model test was performed for each *F. excelsior* sample independently.

### Gene ontology enrichment in differentially methylated homeologs

The R package ‘TopGO’ [58] was used in R v3.1.2, to analyze the enrichment of Gene Ontology (GO) terms in the set of differentially methylated homeologs. Fisher’s exact test with the ‘weighted’ method was used in TopGO, which weights the enrichment score of each node based on that of its neighbours [59]. A comparison was made of the differentially methylated homeologs against the entire set of *F. excelsior* homeologs (all duplicated gene pairs with Ks values between 0.2–0.4 and 0.5–0.8).

### Methylation differences between isogenic samples

We used three different methods to cluster all the samples into groups based on their methylation profile; Principal Components Analysis (PCA), hierarchical clustering, and Pearson’s correlation coefficient. All three methods used a core set of 400,000 cytosines positions that were covered by at least 10 reads in all samples. By using this smaller set of high quality positions, run-time and memory usage was reduced and missing data points were excluded, whilst still retaining a representative sample of genome-wide cytosines. Methylation values were once again adjusted using the logit transformation for PCA and hierarchical clustering (‘logit’ function in R). For PCA, ‘prcomp’ was used with default parameters. To obtain a distance matrix, ‘dist’ function was used with ‘method = “Euclidean”’, and the hierarchical clustering was performed using ‘hclust’ with ‘method = “complete”’. A correlation matrix was made using non-adjusted methylation values with N-DMP positions removed, using the ‘cor’ function in R with ‘method = “pearson”’.

### Methylation and ADB susceptibility

We used two methods to investigate possible links between DNA methylation and ADB susceptibility. Firstly, we investigated methylation patterns in twenty genes that were already found to have expression levels associated with ADB [41]. We calculated the weighted methylation values across these twenty genes for each sample and then tested for differential methylation between the two groups (low versus high susceptibility) using a t-test for each gene.

Secondly, we searched for Differentially Methylated Regions (DMRs) between the high and low susceptibility samples, using metilene V0.2–6 [52]. Metilene detects DMRs between groups of samples using a segmentation algorithm. The input was a matrix of cytosine positions and their methylation values for each sample, with group membership identified within the sample names. Missing data points (read depth < 4×, or those positions filtered out during data QC) were filled with a dash (‘-’) character. We ran separate analyses for each cytosine context, and excluded the low coverage trees for this analysis (F.exc 40-1, F.exc 35-5, F.exc 33-5) so as not to skew methylation values for their group. Parameters ‘-X 5 -Y 6’ (minimum 5 samples from group 1 and 6 from group 2) were used to allow for samples with uncovered regions. We re-ran this analysis a second time, including *F. mandschurica* samples as low-susceptibility samples with the ‘-Y’ parameters increased to 9, due to the addition of the three samples.

## Additional files

Additional file 1: WGBS yield, genome coverage, methylation level and bisulphite conversion rate for twenty ash samples. (PDF 47 kb)

Additional file 2: List of homeologs that are significantly differentially methylated in multiple samples. (PDF 59 kb)

Additional file 3: GO term enrichment in differentially methylated homeologs, compared to the entire set of homeologs. Data separated into Biological Process (BP), Cellular Component (CC), and Molecular Function (MF) tabs. (XLSX 211 kb)

Additional file 4: Genomic positions with most effect on separation of the two *Fraxinus* species in PCA, based on loading along PC1. (PDF 34 kb)

Additional file 5: Average weighted methylation levels in twenty genes known to be associated with ADB susceptibility, for all low and high susceptibility samples (PDF 96 kb)

Additional file 6: DMRs in each cytosine context between high and low susceptibility samples of *F. excelsior*. (XLSX 1771 kb)

Additional file 7: DMRs in each cytosine context between high susceptibility samples of *F. excelsior*, and *F. mandshurica* samples and low susceptibility samples of *F. excelsior*. (XLSX 1149 kb)

Additional file 8: DMRs (and associated genes if present) that obtain a more significant q-value when *F. mandshurica* samples are added into the DMR analysis. (TXT 29 kb)

## Abbreviations

ADB: Ash dieback
BP: Biological Process
C: Cytosine
CC: Cellular Component
C_m_: Methylated cytosine
C_u_: Unmethylated cytosine
DMR: Differentially methylated region
G: Guanine
GO: Gene Ontology
H: Adenine, thymine or cytosine
K_s_: Synonymous substitutions per synonymous site
MF: Molecular function
*MscS*: Mechanosensitive ion channel domain-containing protein
N-DMP: Non-Differentially Methylation Position
PCA: Principal Components Analysis
RRBS: Reduced representation bisulphite sequencing
TE: Transposable elements
WGBS: Whole genome bisulphite sequencing
WGD: Whole genome duplication
