# Supplementary material for: Genome-wide epigenetic variation among ash trees differing in susceptibility to a fungal disease
Source: BMC Genomics. 2018 Jun 28;19:502. doi: 10.1186/s12864-018-4874-8 (PMC6022711; doi:10.1186/s12864-018-4874-8)
Supplement: Supplementary file 1 — WGBS yield, genome coverage, methylation level and bisulphite conversion rate for twenty ash samples. (PDF 47 kb) [file 12864_2018_4874_MOESM1_ESM.pdf]

Sample	Million raw reads	Million reads post-QC	Million mapped reads	Million cytosines covered >3 reads	Conversion Rate (%)	Mean Methylation (%)		
						CG	CHG	CHH
F. mand 1	114.5 (13.0x)	82.2 (8.5x)	61.1 (7.7x)	27.4	98.77	75.16	60.46	16.47
F. mand 2	115.8 (13.2x)	85.8 (8.8x)	65.6 (8.3x)	34.4	98.55	74.60	57.77	13.72
F. mand 3	91.6 (10.4x)	63.4 (6.5x)	46.9 (5.9x)	20.8	98.56	76.58	62.93	15.46
**F. mand pooled**	**322.0 (36.6x)**	**231.3 (23.8x)**	**173.6 (22.0x)**					
F. exc 27-1	87.3 (9.9x)	61.7 (6.3x)	50.2 (6.4x)	24.9	99.07	82.07	68.03	18.45
F. exc 27-2	104.7 (11.9x)	74.0 (7.6x)	61.4 (7.8x)	29.1	99.12	82.55	68.04	16.87
**F. exc 27 pooled**	**192.1 (21.8x)**	**135.7 (13.9x)**	**111.6 (14.1x)**					
F. exc 33-1	87.5 (9.9x)	58.5 (6.0x)	49.1 (6.2x)	24.4	99.05	82.63	67.92	17.07
F. exc 33-2	124.9 (14.2x)	85.5 (8.8x)	74.3 (9.4x)	43.3	99.08	81.05	62.64	13.09
F. exc 33-3	111.7 (12.7x)	74.8 (7.7x)	65.0 (8.2x)	35.2	99.27	81.09	64.19	14.51
F. exc 33-4	119.6 (13.6x)	82.4 (8.5x)	68.8 (8.7x)	33.3	98.92	81.77	65.70	17.13
F. exc 33-5	69.8 (7.9x)	46.9 (4.8x)	38.5 (4.9x)	18.4	98.97	83.66	70.55	22.66
**F. exc 33 pooled**	**513.5 (58.4x)**	**348.0 (35.8x)**	**295.7 (37.4x)**					
F. exc 35-1	116.6 (13.3x)	81.2 (8.4x)	67.1 (8.5x)	31.9	98.98	82.20	66.46	18.06
F. exc 35-2	128.8 (14.6x)	90.4 (9.3x)	74.4 (9.4x)	34.2	99.15	81.55	65.45	17.97
F. exc 35-3	112.0 (12.7x)	81.0 (8.4x)	68.4 (8.7x)	31.9	99.26	80.47	63.27	14.04
F. exc 35-4	106.4 (12.2x)	70.4 (7.3x)	57.4 (7.3x)	23.4	99.29	79.73	64.75	16.17
F. exc 35-5	61.2 (7.0x)	39.4 (4.1x)	31.8 (4.0x)	13.3	99.17	81.02	68.79	19.54
**F. exc 35 pooled**	**524.9 (59.7x)**	**362.5 (37.3x)**	**299.2 (38.0x)**					
F. exc 40-1	41.2 (4.7x)	26.1 (2.7x)	21.8 (2.8x)	10.5	99.03	83.45	71.28	18.02
F. exc 40-2	120.4 (13.7x)	86.4 (8.9x)	70.7 (8.9x)	33.8	99.05	81.54	65.80	19.21
F. exc 40-3	121.0 (13.8x)	83.1 (8.5x)	64.2 (8.1x)	32.3	98.77	81.58	65.18	17.07
F. exc 40-4	113.6 (12.9x)	79.6 (8.2x)	65.0 (8.2x)	32.1	98.59	82.25	66.39	17.14
F. exc 40-5	103.8 (11.8x)	73.4 (7.5x)	60.1 (7.6x)	28.1	99.14	82.41	67.97	20.31
**F. exc 40 pooled**	**500.1 (56.8x)**	**348.6 (35.8x)**	**281.8 (35.7x)**					
**All F. exc pooled**	**1730.6 (196.7x)**	**1194.8 (122.8x)**	**988.3 (125.2x)**	**164.4**	**99.23**	**76.18**	**52.04**	**13.92**

WGBS yield for twenty ash samples. Approximate 880 Mbp genome coverage values in brackets. Coverage value in mapped reads columns shows coverage of 718 Mbp non-N genome. Conversion efficiency for each sample was used in separate binomial tests as described in Methods. Mean Methylation (%) describes percentage of cytosines in each sequence context that are methylated.
