# Supplementary material for: Genome-wide epigenetic variation among ash trees differing in susceptibility to a fungal disease
Source: BMC Genomics. 2018 Jun 28;19:502. doi: 10.1186/s12864-018-4874-8 (PMC6022711; doi:10.1186/s12864-018-4874-8)
Supplement: Supplementary file 2 — List of homeologs that are significantly differentially methylated in multiple samples. (PDF 59 kb) [file 12864_2018_4874_MOESM2_ESM.pdf]

Most consistently differentially methylated homeologs. Counts depict how many times the pair is present in the lowest ten p-values across all *F. excelsior* samples. Note that the table is split into CG, CHG, and CHH contexts (CHG and CHH continued onto next page).

Gene 1	Gene 2	Count	Function	GO terms
**CG**				
150950	150960	13	FAD-linked oxidase, N-terminal	Several, e.g. GO:0050660 (MF: Flavin-adenine dinucleotide binding), GO:0006950 (BP: response to stress), GO:0006040 (BP: amino sugar metabolic process)
133990	299030	10	kinesin-like protein, atp-binding	Several, e.g. GO:0007018 (BP: microtubule based movement), GO:0008569 (MF: ATP-dependent microtubule motor activity, GO:0005871 (CC: kinesin complex)
146520	329760	9	dihydrolipoyllysine-residue acetyltransferase component of pyruvate dehydrogenase mitochondrial-like	Several, e.g. GO:0009941 (CC: chloroplast envelope), GO:0022626 (CC: cytosolic ribosome), GO:0016746 (MF: transferase activity)
227480	280260	6	mfp1 attachment factor 1-like	GO:0005515 (MF: protein binding), GO:0043231 (CC: intracellurar membrane-bound organelle)
285590	398790	5	ring-h2 finger protein	GO:0008270 (MF: zinc ion binding)
169730	353730	5	cationic amino acid transporter	Several, e.g. GO:0003333 (BP: amino acid transmembrane transport), GO:0005886 (CC: plasma membrane)
115990	166400	5	inter-alpha-trypsin inhibitor heavy	GO:0016020 (CC: membrane)
113170	126410	5	anthocyanin 5-aromatic	GO:0047672 (MF: anthranilate N-benzoyltransferase activity), GO:0080167 (BP: response to karrikin (plant growth regulator))
159580	240960	4	laccase 110am multicopper oxidase	Several e.g. GO:0046688 (BP: response to copper ion), GO:0005507 (MF: copper ion binding), GO:0052716 (MF: hydroquinone:oxygen oxidoreductase activity),
250570	373520	4	snrk1-interacting protein 1	GO:0009507 (CC:chloroplast)
**CHG**				
094250	368880	17	atpase splayed	GO:0003677(MF: DNA binding), GO:0005524(MF: ATP binding), GO:0004386 (MF: helicase activity)
133990	299030	17	kinesin-like protein, atp-binding	Several, e.g. GO:0007018 (BP: microtubule based movement), GO:0008569 (MF: ATP-dependent microtubule motor activity, GO:0005871 (CC: kinesin complex)
026950	114060	15	None annotated	GO:0003676(MF: nucleic acid binding), GO:0003723 (MF: RNA binding), GO:0006396 (BP: RNA processing)
307070	375990	14	Zinc finger, PHD-type	GO:0008270 (MF: zinc ion binding)
013250	272830	13	None annotated	No GO terms. IPR 020864 (Membrane attack complex component/perforin (MACPF) domain)
339290	380270	12	FAD-linked oxidase	Several, e.g. GO:0050660(MF: flavin adenine dinucleotide binding), GO:0006979(BP: response to oxidative stress), GO:0009793 (BP: embryo development), GO:0010197 (BP: polar nucleus fusion)
327050	381680	12	cytosolic fe-s cluster assembly factor nbp35	GO:0005524 (MF: ATP binding), GO:0042803 (MF: protein homodimerization activity), GO:0051536(MF: iron-sulfur cluster binding)
155290	378020	11	cytosolic delta subunit	Several e.g. GO:0006457 (BP: protein folding), GO:0046686 (BP: response to cadmium ion), GO:0005524 (MF: ATP binding)
024730	073330	9	regulatory-associated protein of tor 1-like	GO:0009793 (BP: embryo development), GO:0016049 (BP: cell growth)
383510	395700	8	None annotated	GO:0005515 (MF: protein binding), IPR017986 (WD40-repeat-containing domain), IPR006594 (LisH homology motif)

CHH results on next page

Gene 1	Gene 2	Count	Function	GO terms
**CHH**				
030090	095610	15	None annotated	GO:0003755 (MF: peptidyl-prolyl cis-trans isomerase activity), GO:0006457 (BP: protein folding)
055530	207470	13	mediator of rna polymerase ii transcription subunit	GO:0001104 (MF: RNA polymerase II transcription cofactor activity), GO:0016592 (CC: mediator complex)
091230	270880	10	actin associated protein	GO:0009536 (CC: plastid)
208290	254280	10	nucleoprotein tpr	GO:0044699 (BP: single organism process), GO:0044428 (CC: nuclear part), GO:0009987 (BP: cellular process)
105380	292350	10	p-loop containing ntpase domain-containing protein	GO:0005524 (MF: ATP binding), GO:0017111 (MF: nucleoside-triphosphatase activity), GO:0005739 (CC: mitochondrion)
163800	175560	8	udp-n-acetylmuramoyl-l-alanyl-d-glutamate– -diaminopimelate ligase-like	Several, e.g. GO:0007049 (BP: cell cycle), GO:0009658 (BP: chloroplast organisation), GO:0009252 (BP: peptidoglycan biosynthetic process)
024720	073340	7	None annotated	GO:0005622 (CC: intracellular), GO:0006886 (BP: intracellular protein transport)
346190	353990	6	None annotated (LRR-containing)	GO:0016301 (MF: kinase activity), GO:0016491 (MF: oxidoreductase activity), GO:0016310 (BP: phosphorylation)
091760	125860	6	None annotated	GO:0055114 (BP: oxidation-reduction process), GO:0010087 (BP: phloem or xylem histogenesis), GO:0032875 (BP: regulation of DNA endoreduplication),
