# Supplementary material for: Genome-wide epigenetic variation among ash trees differing in susceptibility to a fungal disease
Source: BMC Genomics. 2018 Jun 28;19:502. doi: 10.1186/s12864-018-4874-8 (PMC6022711; doi:10.1186/s12864-018-4874-8)
Supplement: Supplementary file 4 — Genomic positions with most effect on separation of the two Fraxinus species in PCA, based on loading along PC1. (PDF 34 kb) [file 12864_2018_4874_MOESM4_ESM.pdf]

Genomic positions with most effect on separation of the two *Fraxinus* species, based on loading along PC1. Only two positions were within gene regions: Contig2376 position 21815 is within gene 119700, a phospholipid-translocating ATPase, and Contig 8085 position 87294 lies within gene 356660, an RNA recognition motif-containing protein.

Contig	Position	Context	PC1 Loading	Mean methylation *F. mandshurica*	Mean methylation *F. excelsior*
5381	50342	CHH	-0.0127	1.000	0.046
1525	59199	CG	-0.0120	1.000	0.026
3593	6587	CHH	-0.0118	1.000	0.147
2376	21815	CHH	-0.0116	1.000	0.124
2226	58007	CG	-0.0112	1.000	0.037
1525	59192	CG	-0.0107	0.976	0.022
3593	6605	CHH	-0.0102	0.961	0.062
1742	78238	CHH	-0.0100	0.961	0.047
1525	59187	CG	-0.0099	0.970	0.031
5567	17522	CHH	-0.0098	0.910	0.057
345	160178	CHH	-0.0095	1.000	0.290
1019	198545	CHH	-0.0095	1.000	0.162
537	163284	CHH	-0.0093	0.972	0.081
1183	127103	CHH	-0.0089	0.523	0.000
1525	59185	CHG	-0.0086	0.944	0.023
3339	39370	CHH	-0.0085	0.774	0.190
4458	28642	CHH	-0.0084	0.896	0.035
3147	44856	CHH	-0.0083	0.751	0.043
2376	112909	CHH	-0.0081	1.000	0.222
2271	6442	CHH	-0.0081	0.984	0.076
3225	5301	CG	0.0133	0.000	0.985
2238	83939	CHG	0.0133	0.000	0.963
2503	15395	CHG	0.0129	0.000	0.973
3315	36341	CG	0.0127	0.000	0.987
8085	87294	CG	0.0125	0.000	0.974
1546	63329	CHG	0.0124	0.000	0.969
1226	46428	CG	0.0124	0.000	0.983
83324	24258	CG	0.0123	0.000	0.968
87	308463	CHG	0.0122	0.000	0.967
817	74700	CG	0.0116	0.000	0.939
1013	39297	CHG	0.0114	0.000	0.893
3129	72215	CHG	0.0114	0.000	0.930
958	80855	CHG	0.0109	0.000	0.768
5552	6983	CG	0.0108	0.000	0.930
4818	47459	CG	0.0107	0.091	0.967
1417	7761	CHH	0.0105	0.000	0.719
2271	886	CG	0.0105	0.000	0.956
48564	569	CHG	0.0104	0.072	0.983
16	144183	CG	0.0104	0.000	0.936
3829	27916	CG	0.0104	0.321	0.983
