# Supplementary material for: Genome-wide epigenetic variation among ash trees differing in susceptibility to a fungal disease
Source: BMC Genomics. 2018 Jun 28;19:502. doi: 10.1186/s12864-018-4874-8 (PMC6022711; doi:10.1186/s12864-018-4874-8)
Supplement: Supplementary file 5 — Average weighted methylation levels in twenty genes known to be associated with ADB susceptibility, for all low and high susceptibility samples (PDF 96 kb) [file 12864_2018_4874_MOESM5_ESM.pdf]

Average weighted methylation levels in twenty genes known to be associated with ADB susceptibility, for all low and high susceptibility samples. 'PD' is short for 'PREDICTED' in second column. Methylation levels only calculated for genes with at least ten cytosines of the sequence context in question, i.e. some genes only have CHH listed as there were <10 CG and CHG cytosines. ± denotes standard error.

Gene	Top BLASTX hit	Mean weighted methylation		T-test p-value	FDR-corrected p-value
		Low susceptibility	High susceptibility		
298540	PD: calcium-transporting ATPase 9, plasma membrane-type [Erythranthe guttata]	CG:0.57±0.08<br>CHG:0.003±0.0018<br>CHH:0.0047±0.002	CG:0.46±0.06<br>CHG:0.003±0.0015<br>CHH:0.007±0.0036	CG:0.308<br>CHG:0.896<br>CHH:0.503	CG:0.676<br>CHG:0.922<br>CHH:0.744
368850	PD: uncharacterized protein LOC105163554 [Sesamum indicum]	CHG:0.012±0.003<br>CHH:0.0145±0.005	CHG:0.016±0.009<br>CHH:0.022±0.014	CHG:0.701<br>CHH:0.521	CHG:0.841<br>CHH:0.744
261470	soc1-like protein [Olea europaea]	CG:0.898±0.012<br>CHG:0.831±0.014<br>CHH: 0.188±0.012	CG:0.946±0.019<br>CHG:0.886±0.012<br>CHH:0.202±0.021	**CG:0.037**<br>**CHG:0.016**<br>CHH:0.537	CG:0.444<br>CHG:0.288<br>CHH:0.744
251090	PD: MADS-box protein SVP-like isoform X1 [Sesamum indicum]	CHH:0.026±0.009	CHH:0.047±0.015	CHH:0.223	CHH:0.643
048340	PD: MADS-box protein SVP-like [Sesamum indicum]	CG:0.854±0.03<br>CHG:0.512±0.046<br>CHH:0.098±0.009	CG:0.773±0.037<br>CHG:0.521±0.027<br>CHH:0.101±0.015	CG:0.300<br>CHG:0.879<br>CHH:0.848	CG:0.657<br>CHG:0.922<br>CHH:0.922
178910	PD: cinnamoyl-CoA reductase 2 [Sesamum indicum]	CHH:0.122±0.037	CHH:0.078±0.04	CHH:0.442	CHH:0.723
245740	PD: mitochondrial arginine transporter BAC2-like [Solanum pennellii]	CHG:0.129±0.03<br>CHH:0.155±0.034	CHG:0.095±0.016<br>CHH:0.157±0.043	CHG:0.405<br>CHH:0.976	CHG:0.709<br>CHH:0.976
173540	PD: MADS-box protein SVP-like isoform X1 [Nelumbo nucifera]	CG:0.6410.033<br>CHG:0.454±0.029<br>CHH:0.096±0.021	CG:0.697±0.081<br>CHG:0.412±0.07<br>CHH:0.057±0.009	CG:0.455<br>CHG:0.522<br>CHH:0.106	CG:0.723<br>CHG:0.723<br>CHH:0.509
032420	PD: uncharacterized protein LOC105168112 [Sesamum indicum]	CHH:0.101±0.029	CHH:0.192±0.056	CHH:0.131	CHH:0.509
265110	PD: potassium channel KAT3 [Erythranthe guttata]	CHH:0.023±0.007	CHH:0.005±0.003	CHH:0.123	CHH:0.509
262400	PD: serine/threonine-protein kinase AFC2 isoform X1 [Sesamum indicum]	CG:0.595±0.113<br>CHG:0.015±0.009<br>CHH:0.006±0.003	CG:0.513±0.059<br>CHG:0.003±0.003<br>CHH:0.001±0.0008	CG:0.599<br>CHG:0.239<br>CHH:0.112	CG:0.776<br>CHG:0.643<br>CHH:0.509
347350	PD: glucuronoxylan 4-O-methyltransferase 1-like [Sesamum indicum]	CHH:0.0025±0.002	CHH:0.003±0.003	CHH:0.858	CHH:0.922
048360	PD: MADS-box protein SVP-like isoform X1 [Sesamum indicum]	CG:0.772±0.038<br>CHG:0.519±0.052<br>CHH:0.135±0.024	CG:0.790±0.026<br>CHG:0.590±0.021<br>CHH:0.176±0.009	CG:0.737<br>CHG:0.228<br>CHH:0.126	CG:0.86<br>CHG:0.643<br>CHH:0.509
199890	PD: uncharacterized protein LOC105957157 [Erythranthe guttata]	CHH:0.292±0.053	CHH:0.193±0.092	CHH:0.329	CHH:0.657
178920	PD: cinnamoyl-CoA reductase 2 [Sesamum indicum]	CG:0.574±0.085<br>CHG:0.028±0.008<br>CHH:0.018±0.005	CG:0.372±0.028<br>CHG:0.107±0.011<br>CHH:0.014±0.006	CG:0.087<br>**CHG:<0.001**<br>CHH:0.645	CG:0.509<br>**CHG:0.007**<br>CHH:0.806
249100	PD: MADS-box protein SOC1-like isoform X1 [Sesamum indicum]	CG:0.864±0.018<br>CHG:0.579±0.051<br>CHH:0.132±0.013	CG:0.845±0.041<br>CHG:0.456±0.066<br>CHH:0.114±0.011	CG:0.634<br>CHG:0.159<br>CHH:0.399	CG:0.806<br>CHG:0.557<br>CHH:0.709
318210	PD: protein EMBRYONIC FLOWER 1 isoform X1 [Sesamum indicum]	CHG:0.020±0.006<br>CHH:0.006±0.004	CHG:0.008±0.008<br>CHH:0.002±0.001	CHG:0.297<br>CHH:0.338	CHG:0.657<br>CHH:0.657
